# Risk of Stroke in Migrainous Women, a Hidden Association: A Systematic Review

**DOI:** 10.7759/cureus.27103

**Published:** 2022-07-21

**Authors:** Samia E Saddik, Sarah N Dawood, Ahmad M Rabih, Ahmad Niaj, Aishwarya Raman, Manish Uprety, Maria Calero, Maria Resah B Villanueva, Narges Joshaghani, Nicole Villa, Omar Badla, Raman Goit, Lubna Mohammed

**Affiliations:** 1 Internal Medicine, California Institute of Behavioral Neurosciences & Psychology, Fairfield, USA; 2 Pediatrics, California Institute of Behavioral Neurosciences & Psychology, Fairfield, USA; 3 Obstetrics and Gynecology, California Institute of Behavioral Neurosciences & Psychology, Fairfield, USA; 4 Research, California Institute of Behavioral Neurosciences & Psychology, Fairfield, USA; 5 Psychiatry and Behavioral Sciences, California Institute of Behavioral Neurosciences & Psychology, Fairfield, USA; 6 General Surgery, California Institute of Behavioral Neurosciences & Psychology, Fairfield, USA

**Keywords:** stoke, women, articles, narratives, meta-analyses, systematic reviews, observational studies, randomized controlled trials, free full-text articles published within the last five years, english-language

## Abstract

Migraine-a term used to describe a unilateral throbbing headache has shown growing evidence of being linked to different types of strokes-particularly ischemic and hemorrhagic. This study aims to identify and summarize the relationship between migraine and the incidents of stroke in women of child-bearing age. This systematic review was based on the Preferred Reporting Items for Systematic Reviews and Meta-Analyses (PRISMA) guidelines. A search was done using PubMed, the British Medical Journal (BMJ), Cochrane library, Google Scholar, and ScienceDirect databases up until March 15, 2022. Studies were chosen based on the listed eligibility criteria: English-language, observational studies, systematic reviews, articles, and meta-analyses, which included stroke patients and migraine patients, and the possible link between these two conditions.

In addition, quality assessment was done using assessment tools like Scale for the Assessment of Narrative Review Articles (SANRA), Assessment of multiple systematic reviews (AMSTAR), and Newcastle-Ottawa Scale (NOS) criteria. The initial search generated 245 studies. Fourteen studies were included in the final selection - one case-control, four cohort studies, seven systematic reviews with meta-analyses, and two narrative reviews. Strokes-particularly ischemic-were found to be linked to the incidents of migraine in women. The risks of a stroke increased if a woman was a smoker, under 45, and uses oral contraceptives regularly. In addition, the use of nonsteroidal anti-inflammatory drugs (NSAIDs), genetic predisposition, and metabolic dysfunction was linked to increased incidents of hemorrhagic strokes-which proved to be rarer but more fatal due to their serious underlying pathophysiologies.

## Introduction and background

Migraine is the most common complaint of around 80% females, with the main symptom being an extreme throbbing headache mostly radiating to one side. In addition, it could be linked to bouts of nausea and light and sound sensitivity [1]. In this day and age, one in six American women experience migraine headaches [2]. The reported incidence of migraine in females of reproductive age has increased over the last two decades, and therefore, this change helped increase the awareness of the condition [3]. Common triggers for a migrainous attack include but are not limited to emotional stress, caffeine, missing meals, and extreme light changes [4,5]. A migraine aura is a temporary disturbance that usually strikes before migraine symptoms - such as throbbing pain, nausea, and sensitivity to light and sound and tends to occur an hour before the onset of a headache [6].

A stroke happens when something prevents blood supply to the brain or when a blood vessel in the brain leaks [4]. Either way, this causes injury or even death to certain parts of the brain, and it needs very urgent care to prevent permanent damage. A stroke can cause brain injury, disability, or even death [7]. Most strokes are ischemic strokes, meaning that it occurs when blood clots or other particles block a brain vessel; meanwhile, hemorrhagic stroke happens when an artery in the brain leaks [8,9]. The lifetime risk of stroke for women during their menopausal years in the United States is one in five [10]. Common risk factors for an attack of stroke are high blood pressure, diabetes, heart disease and oral contraceptives [11].

Migraine and stroke differ in epidemiology, onset, clinical presentation, prognosis, and treatment [12]. Interestingly, research has pointed toward a possible relationship between migraine and stroke [13,14]. A possible link between these two conditions can prove to be a substantial burden on society [15]. A morbid outcome could occur if a hemorrhagic stroke or ischemic stroke with migraine were misdiagnosed as migraine with aura [16,17]. Few studies have addressed the reasons for an association between migraine and stroke; however, the exact mechanism as to why there is a relationship is still not fully understood [18].

This systematic review aims to outline the potential risks of stroke in migrainous females, as it has been a mystery whether migraine is linked to an increased risk of stroke. Available literature and other systematic review papers on the link between migraine and stroke were reviewed, in addition to pathophysiologies and clinical manifestations of this association.

Methods

This systematic review was influenced by the Preferred Reporting Items for Systematic Reviews and Meta-Analyses (PRISMA) 2020 guidelines [6].

Eligibility Criteria 

Inclusion criteria were added and included the following: English-language, Free Full-Text articles published within the last five years, randomized controlled trials (RCTs), observational studies, systematic reviews, and meta-analyses, narratives, articles, women, stroke, migraine, headache, women of child-bearing age, migraine, migraine with aura, ischemic/hemorrhagic stroke, migrainous infarction, risk factors, vascular disease. Exclusion criteria: Other genders, age group younger than 19, management of stroke, management of migraine, paid articles.

Databases and Search Strategy

The search was done systematically using PubMed, Cochrane library, Google Scholar, and ScienceDirect, and the BMJ databases. The last date of search for the databases was March 15, 2022. The searches used in the process were selected based on the keywords used previously and through Medical Subject Headings (MeSH). Search strategy is shown in Table 1.

**Table 1:** The strategy of the bibliographic search in databases with their corresponding filters. BMJ: British Medical Journal

Databases	Keywords	Search Strategy	Filters	Search Results
PubMed	Headache, migraine, women, stroke, ischemic stroke, hemorrhagic stroke, brain death, adult women, brain attack, thrombus, embolus	Stroke OR Brain Attack OR Embolus OR Thrombus OR ( "Stroke/epidemiology"[Majr] OR "Stroke/ethnology"[Majr] OR "Stroke/etiology"[Majr] OR "Stroke/physiopathology"[Majr] ) AND Migraine OR Headache OR ( "Migraine Disorders/epidemiology"[Majr] OR "Migraine Disorders/ethnology"[Majr] OR "Migraine Disorders/etiology"[Majr] OR "Migraine Disorders/physiopathology"[Majr] ) AND Women OR Females	Free papers, last 5 years, humans, English, females, Adults 19+	70
Cochrane Library	Headache, migraine, women, stroke, ischemic stroke, hemorrhagic stroke, brain death, adult women, brain attack, thrombus, embolus	Stroke OR Brain Attack OR Embolus OR Thrombus OR ( "Stroke/epidemiology"[Majr] OR "Stroke/ethnology"[Majr] OR "Stroke/etiology"[Majr] OR "Stroke/physiopathology"[Majr] ) AND Migraine OR Headache OR ( "Migraine Disorders/epidemiology"[Majr] OR "Migraine Disorders/ethnology"[Majr] OR "Migraine Disorders/etiology"[Majr] OR "Migraine Disorders/physiopathology"[Majr] ) AND Women OR Females	Last two years, English	22
Google Scholar	Headache, migraine, women, stroke, ischemic stroke, hemorrhagic stroke, brain death, adult women, brain attack, thrombus, embolus	Stroke OR Brain Attack OR Embolus OR Thrombus OR ( "Stroke/epidemiology"[Majr] OR "Stroke/ethnology"[Majr] OR "Stroke/etiology"[Majr] OR "Stroke/physiopathology"[Majr] ) AND Migraine OR Headache OR ( "Migraine Disorders/epidemiology"[Majr] OR "Migraine Disorders/ethnology"[Majr] OR "Migraine Disorders/etiology"[Majr] OR "Migraine Disorders/physiopathology"[Majr] ) AND Women OR Females	2017-2022	120
Science Direct	Headache, migraine, women, stroke, ischemic stroke, hemorrhagic stroke, brain death, adult women, brain attack, thrombus, embolus	Stroke OR Brain Attack OR Embolus OR Thrombus OR ( "Stroke/epidemiology"[Majr] OR "Stroke/ethnology"[Majr] OR "Stroke/etiology"[Majr] OR "Stroke/physiopathology"[Majr] ) AND Migraine OR Headache OR ( "Migraine Disorders/epidemiology"[Majr] OR "Migraine Disorders/ethnology"[Majr] OR "Migraine Disorders/etiology"[Majr] OR "Migraine Disorders/physiopathology"[Majr] ) AND Women OR Females	2017-2022, review articles, medicine and dentistry, journal of neurological sciences	21
BMJ	Headache, migraine, women, stroke, ischemic stroke, hemorrhagic stroke, brain death, adult women, brain attack, thrombus, embolus	Stroke OR Brain Attack OR Embolus OR Thrombus OR ( "Stroke/epidemiology"[Majr] OR "Stroke/ethnology"[Majr] OR "Stroke/etiology"[Majr] OR "Stroke/physiopathology"[Majr] ) AND Migraine OR Headache OR ( "Migraine Disorders/epidemiology"[Majr] OR "Migraine Disorders/ethnology"[Majr] OR "Migraine Disorders/etiology"[Majr] OR "Migraine Disorders/physiopathology"[Majr] ) AND Women OR Females	2017-2022, open access articles	12

All references were collected using Microsoft Excel (Microsoft Office Professional Plus 2019, Microsoft Corp., Redmond, WA) for duplicate removal. The records were reviewed based on the titles and abstracts, excluding irrelevant studies. Due to the few systematic reviews, meta-analyses, and narrative reviews in the area, the writers chose to include them in the study.

Risk of Bias in Individual Studies

The articles were checked for quality and risk of bias using the Newcastle Ottawa Scale (NOS) for cohort studies; Systematic reviews and Meta-analyses used Assessment of Multiple Systematic Reviews (AMSTAR), and for Narrative reviews, we used the Scale for the Assessment of Narrative Review Articles (SANRA). 

Results

Study Selection and Quality Assessment

**Figure 1:**
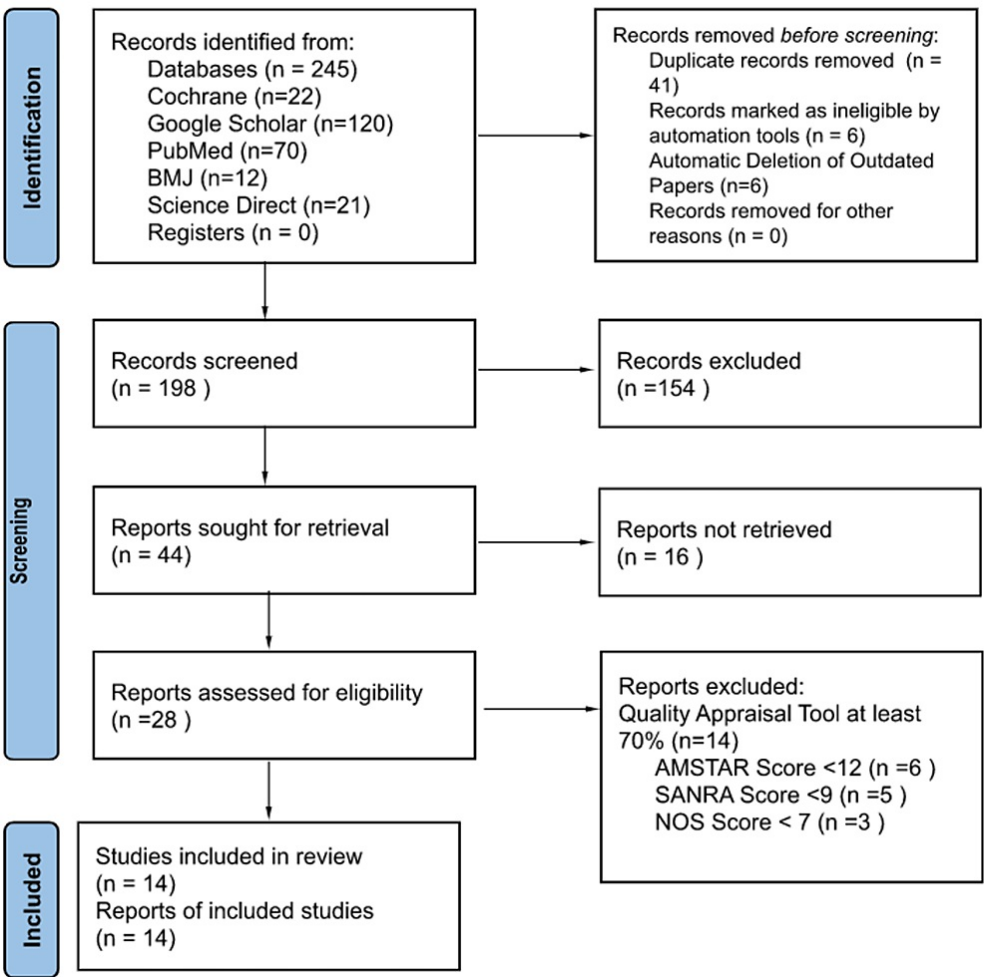
Figure 1: Flow chart of the study search selection. BMJ: British Medical Journal, AMSTAR: Assessment of Multiple Systematic Reviews 2, SANRA: Scale for the Assessment of Narrative Review Articles, NOS: Newcastle Ottawa Scale

The database search showed 245 potentially relevant titles from five different databases, as mentioned above. Forty-one titles were removed in total as duplicates, six marked ineligible, and six papers were removed because they were outdated, with 198 records retained. These articles were screened for relevant information, and 154 were excluded as a result. Therefore, 44 reports remained and were retrieved, and 16 reports were not retrieved. Finally, a quality assessment was done, and 14 studies with a score of greater than 70% were accepted in the review process. These were one case-control, four cohort studies, seven systematic reviews with meta-analyses, and two narrative reviews. A diagram showing the screening outline is shown in Figure 1.

Study Characteristics

The main characteristics of the Systematic Reviews, Cohort studies, Case-Controls, and Narrative Reviews are shown in Table 2. 

**Table 2:** Main characteristics of the Cohort studies, Case-control studies, Systematic reviews, and Literature Reviews accepted in the review. I: Inclusion, E: Exclusion, IS: Ischemic stroke, CeAD: Cervical artery dissection, MA: Migraine with aura, MO: Migraine without aura, HC: Hormonal contraceptives

First Author, Year	Study Type	Disease	Inclusion/Exclusion Criteria	Gender, Age	Outcomes
Knol, 2020 [19]	Narrative Review	Migraine genetics	-	-	Migraine risk is associated with alterations in cerebral hemodynamics.
Rohmann, 2020 [20]	Cohort-Study	Migraine & Mortality	I: Migraine, Headache, Mortality, Women, Epidemiology	Women	In this large prospective study of women, we found no association between non-migraine headaches or migraine and all-cause mortality.
Lee, 2019 [21]	Cohort-Study	Ischemic Stroke & Migraine	I: 2002-2013, women	Young women	Migraine is associated with an increased risk of ischaemic stroke, but not haemorrhagic stroke.
Gill, 2020 [22]	Cohort-Study	Vascular events & Migraine	-	18-64	Recent rates of vascular disease in patients with migraine.
De Giuli, 2017 [23]	Cohort-study	Migraine & Cervical Artery Dissection	-	18-45	In patients with IS aged 18 to 45 years, migraine, especially migraine without aura, is consistently associated with CeAD.
Tietjen, 2018 [24]	Case-Control Study	Migraine & Vascular Disease	-	30-60, women	Endothelial activation elevated biomarkers of hypercoagulability and inflammation and is associated with migraine, particularly in women.
Oie, 2020 [7]	Systematic review	Migraine & Stroke	I: From 2011 up to March 2019. ‘migraine AND (stroke OR ischemic OR hemorrhagic OR cerebrovascular disease OR brain infarct OR transient ischaemic attack OR intracranial hemorrhage OR subarachnoid hemorrhage)’A subsequent exclusion process was done based on language	>60+	Changes in cerebrovascular reactivity and cerebral ischaemic threshold in people with MA may also increase the risk of ischaemic stroke.
Hassan, 2021 [9]	Systematic Review	Migraine & Stroke pathways	I: Migraine-associated vasospasm, cortical spreading depression, migraine-related stroke, hemorrhagic stroke, ischemic stroke, migraine with aura, stroke, migraine	-	Migraines, particularly migraines with aura, should be considered an important risk factor for ischemic stroke.
Zhang, 2017 [11]	Systematic Review	Migraine & Stroke	I: Migraine AND (stroke OR ischemic OR hemorrhagic OR cerebrovascular disease OR brain infarct OR transient ischaemic attack OR intracranial hemorrhage OR subarachnoid hemorrhage)	-	Migraine is associated with an increased risk for stroke^, ^although the etiology of stroke in migraineurs remains unclear.
Sacco, 2017 [13]	Systematic Review	Stroke & Contraceptives	I: “Migraine” AND (contraceptive OR estrogen) AND (vascular OR stroke OR “myocardial infarction” OR angina OR “coronary artery disease” OR “coronary heart disease” OR “venous thrombosis”)	Women	Evidence addressing the risk of ischemic stroke associated with the use of HCs is generally poor.
McKinley, 2021 [14]	Systematic Review	Ischemic Stroke & Migraine	I: Migraine, Myocardial infarction, Coronary revascularization, Ischemic stroke	60+	Older adults with migraine are at increased risk for ischemic stroke.
Linstra, 2021 [15]	Systematic Review	Sex differences in stroke	I: Sex differences, migraine, stroke outcome, stroke subtype, cardiovascular risk factors	-	Possible sex differences in the pathophysiology underlying the migraine–stroke association
Daghlas, 2022 [18]	Narrative Review	Migraine & Cervical dissection	I: Types of strokes, migraine, types of migraine	-	Among all pairs of disorders, the genome-wide genetic correlation was observed only between CeAD and migraine, particularly MO.
Van der Weerd, 2021 [16]	Systematic Review	Sex differences in Migraine	E: male I: female, risk factor, migraine, coagulation, plasma, serum	Females	Sex differences exist in the activation of the hemostatic system in ischemic stroke.

Of the 14 studies accepted in the review, seven articles were systematic reviews, while four were cohort studies. In addition, one was a case control, and two were literature reviews. Moreover, the Systematic Reviews and Literature Reviews further outlined the positive correlation between stroke incidents and migraine in women. In addition, the cohort studies suggested that there was an increase in vascular incidents among stroke sufferers in several prospective studies, including women [20-23]. Finally, a case-control study showed that endothelial activation caused an elevation in biomarkers of hypercoagulability and inflammation and is therefore associated with migraine, particularly in women [24]. Table 2 above shows these findings.

## Review

Discussion

In this section, an overview of stroke and migraine types, pathophysiology, epidemiology, and associations will be addressed. A short statement regarding the limitations of this study will also be noted at the end.

Migraine Anatomy

A plexus originating from the ophthalmic branch of the trigeminal nerve acts as the main culprit in the pathogenesis of migraines [7]. A typical migraine attack mainly consists of four phases, which may vary among different persons. These phases include the prodrome, aura, headache, and postdrome [8]. A prodrome lasts anywhere from hours to days and typically affects concentration and vision. Next, the aura phase starts an hour before the onset of headache, and common symptoms include flashing lights and blurry vision. Moreover, the headache phase lasts anywhere from one to 72 hours and usually has a unilateral, throbbing pain pattern associated with neck pain and nausea. Finally, the postdrome phase follows the headache, and the common symptoms include fatigue and altered mood. Table 3 demonstrates the difference between all stages and was made by author Samia.

**Table 3:** Phases of a Migraine

Stage	Timing	Symptoms
Prodrome	Hours to days before onset of headache	Concentration problems, depression, and photophobia
Aura	Hour before onset of headache	Flashing lights, wavy lines, blurry vision
Headache	Lasts one to 72 hours	Unilateral pain, neck pain, photophobia, nausea
Postdrome	Follows immediately after the headache	Fatigue, moodiness, feeling of well-being

Epidemiology of a Migraine

**Figure 2:**
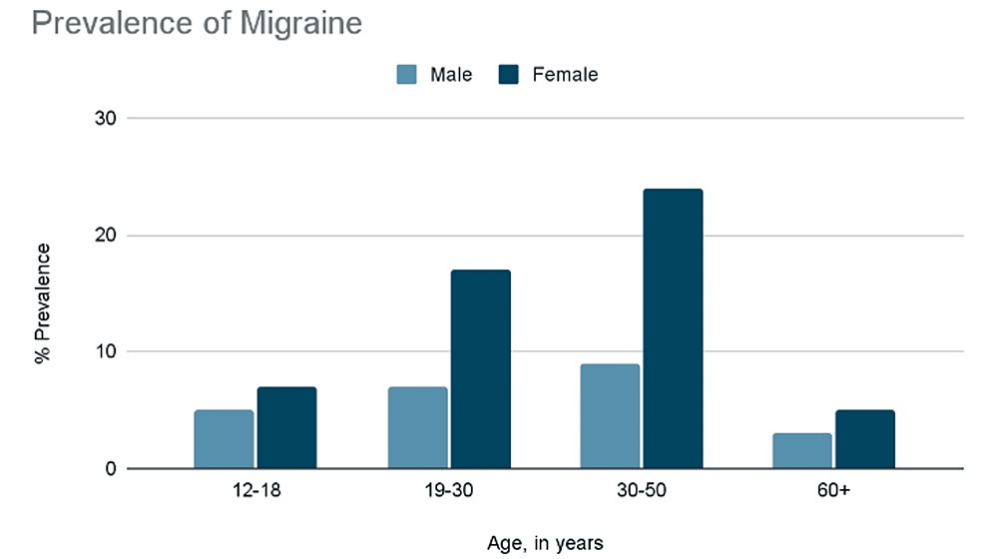
Prevalence of Migraine

It has become clear that the susceptibility to migraines is indeed inherited [11]. The molecular genetics for migraine was found by missense mutations in the α1A subunit voltage-gated calcium channel on chromosome 19 and were later linked to familial hemiplegic migraine [9]. In addition, migraines tend to occur in 91% of men and 96% of women, and six percent of men and 18% of women (one-year prevalence) [10], and are, in fact, the most prevalent in the third decade of life and in lower socioeconomic groups [11]. The prevalence in teenagers is around five to nine percent and increases as the patient ages. For example, in young adults and middle age, it ranges from 18% to 25% prevalence and seems to decline to around five percent as the patient approaches 60 years of age. Moreover, it is linked to cardiovascular disease, psychiatric conditions, and sleep disorders and is considered the second most disabling condition in the world [12]. Figure 2 was influenced by a study published in 2019 [13] and summarizes the prevalence findings among different age groups and genders [13].

Pathophysiology of a Migraine

According to some researchers, inflammation of the dura mater could result in possible migraine pain [13]. Moreover, some structural changes were noted in the dura mater directly after trigeminal excitation. The changes observed included mast cell degranulation, postcapillary venule alterations, and platelet aggregation [14]. It is globally known that such changes in the dura may be the reason a migraine happens; however, there is no clear evidence that this change itself is enough to cause the pain or if it works together with other promoters [14].

Moreover, it was found that electrical stimulation of the trigeminal ganglion led to an increase in cerebral blood flow [15]. This happens through a series of pathways that release vasoactive intestinal peptides (VIP). In addition, the cerebral blood vessels that receive VIP-ergic innervation are mostly anterior rather than posterior, thus adding to this region's susceptibility and why the aura originates posteriorly [15].

Anatomy of Stroke

A stroke may occur when there's an obstruction of blood flow and oxygen to the brain and could be due to a blockage in a blood vessel or if a vessel ruptures [16]. In addition, different types of strokes exist; one of them is ischemic stroke, which is the most common type, and the other one is hemorrhagic stroke, the less common type [17]. Our brain needs a constant supply of oxygen, and when there’s a lack of it, it quickly deteriorates and causes damage to the deprived area. As a result, the symptoms of a stroke may differ depending on the location. Moreover, the signs of a stroke can include sudden loss of balance, sudden loss of vision, facial weakness, arm weakness, and altered speech [16]. Table 4 outlines the main signs and symptoms of a stroke, and they can be remembered by the mnemonic BE FAST [18]. It's important to act quickly and become oriented with these signs as they can be life-saving and ensure the best possible treatment and outcomes.

**Table 4:** Signs and Symptoms of Stroke The Table was created by author Samia

Letter	Sign/Symptom
B	Balance: Sudden loss of balance
E	Eyes: Sudden loss of vision
F	Face: Facial weakness
A	Arms: Arm weakness/numbness
S	Speech: Altered speech
T	Time: Act fast

Epidemiology of Stroke

Roughly 800,000 primary strokes occur annually in the U.S., with primary strokes making up around 600,000 [19]. Ischemic strokes make up around 87%, 10% are hemorrhagic strokes, and three percent constitute subarachnoid hemorrhages. It is estimated that primary hemorrhages make up a larger number of all strokes, ranging from 10%-25% [19]. Patients that are of Asian, African, and Latin American descent are at an increased risk and more susceptible to primary hemorrhages than patients of European descent [19]. Moreover, primary hemorrhages make up 10%-17% of all strokes in the West and 25% in Asia [20].

**Figure 3:**
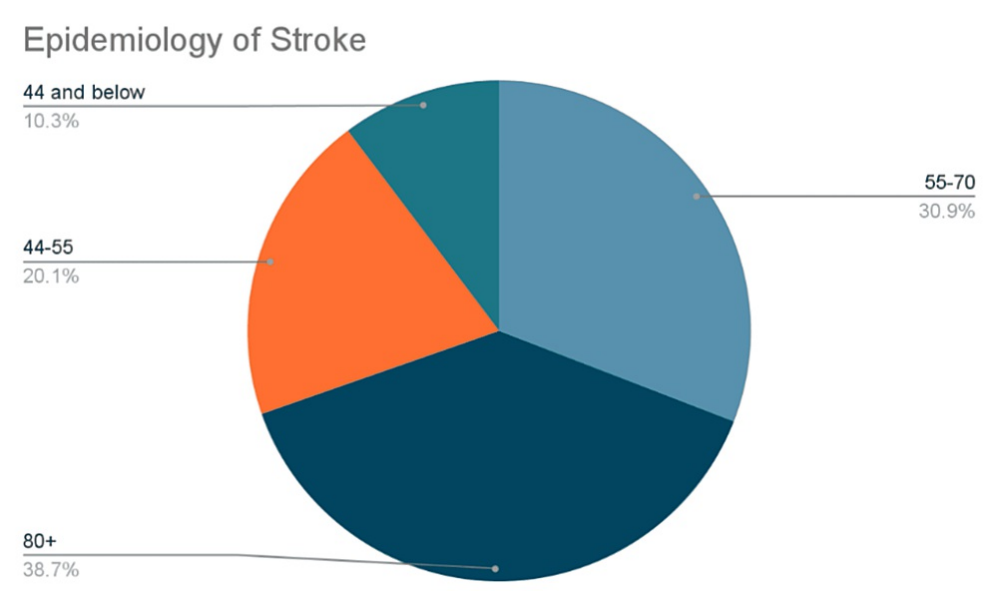
Epidemiology of Stroke

The incidence increases with age, doubling after age 50 [20]. Among middle-aged adults, the incidence is 30 to 120 of 100,000 per year, and for the elderly, it is 670 to 970 of 100,000 per year [20]. Children can also suffer from strokes; however, the incidence is significantly lower. To put it into perspective, patients 44 years and younger have around a 10% chance of getting a stroke, between 44 and 55 around 20%, 55 to 70 around 31%, and the most would be around 40% in patients above 80 years old. Figure 3 was influenced by a study published in 2010 [21] and summarizes the epidemiological findings.

Pathophysiology of Stroke

There are mainly two types of stroke; Ischemic stroke is due to impaired blood and oxygen supply to the brain either by a thrombus or embolus; hemorrhagic stroke is caused by leaking blood vessels [21].

Ischemic strokes make up the majority of strokes in patients; meanwhile, hemorrhagic strokes make up the rest [22]. In the case of a thrombus, blood flow is prevented by plaque build-up or atherosclerosis. The build-up of plaque will eventually constrict the blood vessels and form clots, resulting in an ischemic stroke [22,23]. Following a stroke, brain necrosis quickly happens by plasma membrane impairment, organelle swelling, and eventual loss of function. In addition, inflammation, loss of homeostasis, acidosis, increased calcium levels, and free radical-mediated toxicity adds to the pathophysiology [24].

Hemorrhagic strokes make up around 10%-15% of all strokes but have the highest mortality rates [24]. In this type of stroke, excessive stress in the brain and internal injuries cause blood vessels to rupture-like in hypertension [22]. It is further divided into an intracerebral and subarachnoid hemorrhage. The most common reasons for intracerebral hemorrhage are hypertension, injury to vasculature, and anticoagulants [23,24]. In subarachnoid hemorrhage, blood accumulates in the subarachnoid space due to injury or cerebral aneurysm.

Association between Stroke and Migraine

Patients with migraine have an elevated risk of ischemic stroke, according to many studies [23,24]. However, new research in large cohorts has re-examined the link between migraine and vascular events in recent years, and a comprehensive systematic review and meta-analysis published in 2009 [23] found a link between migraine with aura and ischemic stroke. The risk of ischemic stroke appears to be highest among smoking women under 45 who use oral contraceptives [24,25]. A migraine aura or a brief ischemia event can cause new migraines in elderly persons. After a first attack, clinical differentiation might be difficult, especially in elderly individuals with vascular risk factors [26].

Migraine, particularly migraine with aura (MA), has been related to an elevated risk of ischemic stroke in several studies, including five meta-analyses [26,27]. When compared to migraine-free adults, the possibility of an ischemic stroke in those with MA is doubled. It's unclear if migraine without aura (MO) increases the risk of ischemic stroke. Women under 45 years, who use oral contraceptives, and smokers had a larger link between migraine and ischemic stroke than males [28,29]. Men have a three-fold lower prevalence of migraine than women, so the link is less certain. Active migraine sufferers and those who have a higher frequency of attacks have a higher chance of suffering an ischaemic stroke; however, proof of migraine severity and chances of stroke remains unclear [30]. Active migraine attacks and a higher frequency of attacks raise the probability of an ischemic stroke incident, but the link between migraine intensity and the risk of stroke is still unknown.

It's difficult to tell the difference between a migraine aura and a transient ischemic attack (TIA), even for expert neurologists [30]. This is especially true for people over 60 years old, who are more prone to have TIAs, whereas MA episodes are likely to become unusual since they are not always followed by a migraine headache [31]. The most significant distinction between TIA and MA is the quick onset of symptoms in the presence of vascular risk factors, as opposed to MA, which has a more gradual development of symptoms [32,33]. Although not well studied, the chances of TIA seem to be increased in people who have migraine with aura but not in those with migraine without aura. The misdiagnosis of migraine aura as TIA might be a stumbling block to a thorough investigation of this link [34].

Any cerebrovascular event can cause a migraine-like attack, which can lead to the misdiagnosis of stroke as a 'complicated migraine.' [30,31]. The increased frequency of migraine aura in older ages may be the expression of certain stroke risk factors like hemosiderin deposition and arterial emboli rather than the aura itself [35].

A link between migraine and hemorrhagic stroke is possible; while some studies have found no link, there is growing evidence that it is especially in women under 45 years old [33,34]. Although some studies have revealed an elevated risk in persons with MA, there is not enough data to conclude that the chances of a hemorrhagic stroke is increased in persons with migraine. While women under 45 may have an increased chance of a hemorrhagic stroke, the overall association remains unknown [26-28].

There are various ideas on what causes hemorrhagic strokes in migraine sufferers. First, migraineurs have a lower quantity of circulating progenitor endothelial cells [24,25]. This, together with a change in artery wall structure found in migraine sufferers, can provide a favorable environment for hemorrhagic strokes [36]. Second, vascular diseases such platelet dysfunction, hypertension, and high cholesterol levels are all risk factors for migraines, as well as hemorrhagic and ischemic strokes [37]. Another method involves a common risk factor: nonsteroidal anti-inflammatory medications (NSAIDs) [31]. When those who use NSAIDs for migraine pain management have ten or more headache days per month, NSAIDs become a contributor to migraine development rather than a preventative component. Furthermore, NSAIDs, particularly ibuprofen, are linked to an increased risk of stroke, making them a likely contributing factor in hemorrhagic strokes in migraine sufferers [37]. Mechanisms linking migraines to ischemic strokes have also been heavily hypothesized. The increasing hypoperfusion and decrease in cerebral blood flow that happens with migraine is one probable cause [36,37].

The mechanism that causes this hypoperfusion is known as "spreading depression" [38] , which is characterized by a significant decrease in potential generating activity within gray matter neuronal membranes that spreads across the cortex at a rate similar to that seen with the progression of migraine symptoms. Vasospasm is another probable cause of cerebrovascular hypoperfusion [39]. This relationship has been proven in several case studies. One study found that a 10 year old child with migraines had vasospasm in the left middle and posterior cerebral arteries [39]. In addition, it was discovered that a lady who had an intense migraine was followed by a series of strokes that resulted in her death [40]. She didn't have any vascular or hematologic risk factors, and she was not taking any medications. Even after ruling out any potential mimicry, arterial vasoconstriction has been seen in numerous additional studies.

Increased concentration and activity of many intravascular procoagulant factors in migraineurs further supports the link between migraines and strokes [40,41]. Antiphospholipid antibodies are one of them, and they promote coagulation by enhancing the affinity of phospholipid complexes for other phospholipids and the cell surface. In addition, they have the ability to activate platelets. Homocysteine, especially the methylenetetrahydrofolate reductase (MTHFR) C677T genotype, is another procoagulant observed in higher amounts in migraineurs and is linked to an increased risk of stroke. The von Willebrand factor is another procoagulant implicated in migraines linked to stroke (vWF) [42]. In addition to vWF, higher amounts of endothelin-1, a strong vasoconstrictor, and prothrombin 1.2, the cleavage product of prothrombin, have been documented in investigations [42]. Prothrombin 1.2 is a biomarker of thrombosis, and it is thought to be a sign of hypercoagulability.

**Figure 4:**
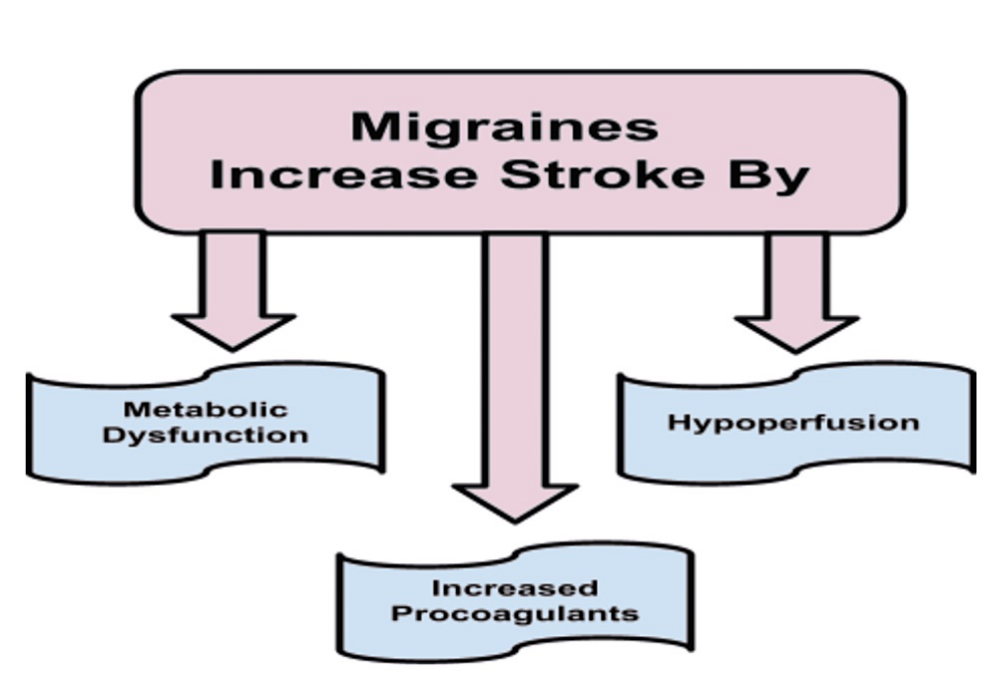
Etiologies of Migraine and Stroke

Figure 4 summarizes the possible etiologies behind migraines and strokes and was created by author Samia. 

Limitations

The limitations of this paper could be summarized as follows; not enough databases were searched as some of them were paid; therefore, only free-full text papers were used. In addition, studies before 2017 were not included as a maximum of five-year old papers were only sought. Moreover, this paper was mainly targeted at the female adult population, and other genders or younger age groups were not taken into consideration. Other interesting topics that could be touched upon are the relationships between migraines and stroke in men, as a small percentage of men also experience migraine attacks in patients with previous histories of CVS disease and the effect of migraine medication on the risk of stroke.

## Conclusions

Overall, the studies that were included in this review prove that there is an evident association between migraine incidents and stroke. This is based on the various literatures that were reviewed across different databases, and clinical trials. In particular, migraines were more closely related to ischemic strokes rather than hemorrhagic, according to statistics, and several risk factors may contribute to this-including the use of oral contraceptives, NSAIDs, smokers, women under the age of 45, and genetics. However, hemorrhagic stroke incidents have proven to be more fatal in women younger than 45 years old, as the mechanism reveals a more serious underlying etiology. Future proposals concerning this study include exploring more studies, like cohorts, studies with larger sample sizes, including the male population in the clinical trials, and younger age groups. In addition, paid databases should be explored as certain data may prove more useful in proving this association. Moreover, these recommendations are made to further explore the dynamic between strokes and migraine in different populations, genders, and age groups. And possibly alert the physicians about migraine misdiagnosis as many early strokes appear to be missed therefore, leading to a poorer prognosis.
